# Successful capture of *Toxocara canis* larva antigens from human serum samples

**DOI:** 10.1186/s13071-015-0875-5

**Published:** 2015-05-08

**Authors:** Aarón Rodríguez-Caballero, Mario Noé Martínez-Gordillo, Yolanda Medina-Flores, María Edith Medina-Escutia, Antonio Meza-Lucas, Dolores Correa, Silvia Caballero-Salazar, Martha Ponce-Macotela

**Affiliations:** Laboratorio de Parasitología Experimental, Instituto Nacional de Pediatría, Insurgentes Sur No. 3700-C, Colonia Insurgentes Cuicuilco. Delegación Coyoacan, México D.F, 04530 México; Laboratorio de Anticuerpos Monoclonales, Instituto de Diagnóstico y Referencia Epidemiológicos, Calle Francisco P Miranda No. 177. Col. Unidad Lomas de Plateros, Delegación Álvaro Obregón, México D.F, 01480 México; Laboratorio de Pruebas Rápidas, Instituto de Diagnóstico y Referencia Epidemiológicos, Calle Francisco P Miranda No. 177. Col. Unidad Lomas de Plateros, Delegación Álvaro Obregón, México D.F, 01480 México; Laboratorio de Inmunología Experimental, Instituto Nacional de Pediatría, Insurgentes Sur No. 3700-C, Colonia Insurgentes Cuicuilco. Delegación Coyoacan, México D.F, 04530 México

**Keywords:** *Toxocara canis*, *Larva migrans*, Antigen capture

## Abstract

**Background:**

*Toxocara canis* is a nematode that parasitizes dogs, while humans are paratenic hosts. When humans are infected the migrating larvae damage the liver, lungs and even the nervous system. *Larva migrans* diagnosis is based on immunological techniques; however, the commercial immunodiagnostic kits detect anti-*T. canis* antibodies which may cross-react with other parasites, mainly nematodes with extra-intestinal migration. Moreover, antibodies do not necessarily reflect an active infection; so detection and quantification of circulating antigens may provide appropriate and timely information for treatment, which prevents irreversible damage. Here we report the standardization of a monoclonal antibody based antigen capture ELISA to diagnose human toxocariasis without cross-reaction.

**Methods:**

We developed anti-*T. canis* polyclonal antibodies in rabbits and a monoclonal antibody in mouse which did not cross-react with 15 antigens from several parasites. The sandwich ELISA standardization was performed using sera from mice experimentally infected. We tested the method using 29 positive and 58 negative human sera previously typified with a commercial kit, which detects antibodies.

**Results:**

Only 5.0 μg/mL and 10 μg/mL polyclonal antibodies and monoclonal antibody, respectively, were needed in the sandwich ELISA standardization, detecting since 440 pg/mL larva antigens. Nine out of 29 antibody-positive sera were also positive for antigens and no false positive were found. Taking the antibody kit as the reference standard, the sensibility and specificity of the antigen test were 31% and 100%, respectively.

**Conclusions:**

With these tools we established a detection threshold as low as 440 pg/mL antigen. Monoclonal antibody is specific, and did not cross-react with antigens from other parasites. Detection of circulating antigens helps provide appropriate and timely treatment and prevents irreversible damage.

## Background

The migration of *Toxocara canis* larvae is injurious to human beings, because they invade the liver, the lungs or the nervous system [1]. Dogs are definitive hosts, and the parasite successfully infects puppies by uterine, trans-mammary or environmental routes, with prevalence near 100% in some places [2]. In contrast, 12-21% of adult dogs are infected with the parasite [3]. As *Toxocara* females shed an average of 68,000 eggs/day, dogs are an important source of environmental contamination [4,5]. Children are most susceptible to infection with *Toxocara* embryonated eggs due to their playing behavior and their tendency to eat dirt. Humans serve as paratenic hosts and the migrating parasite produces: visceral *larva migrans* (VLM) characterized by hepatic damage and Löffler syndrome with fever, pulmonary inflammatory infiltrate and eosinophilia [6]; ocular *larva migrans* (OLM) which in severe cases leads to eyesight loss [7]; eosinophilic meningo-encephalitis (EME) [8]; and covert toxocariasis (CT) [9]. Currently, *larva migrans* is diagnosed by immunological methods, which detect antibodies against excretion-secretion antigens [10]*.* However, this method has limitations, i.e. there is cross-reactivity with antigens from other parasites [10-12]*.* For treatment purposes it is important to know if there are circulating antigens. There have been few reports that show the capture of *T. canis* larvae excretion and secretion antigens (L_2_TES) as an alternative diagnostic strategy, but with variable results [13-15]. Here, we report the standardization of an ELISA to capture and quantify circulating *Toxocara* antigens to diagnostic human toxocariasis without cross-reaction.

## Methods

### Ethical approval

Protocol was approved by the research and ethic committees of National Institute of Pediatrics. All animal procedures were performed in accordance with the guidelines of the Coordinator Commission of the National Institutes of Health of Mexico (Institutos Nacionales de Salud, NOM-062-ZOO-1999).

### *Toxocara canis* larvae

*T. canis* adults were obtained from the small intestines of puppies euthanized at the Canine Control Centre in Tlalpan, México D.F., as described elsewhere [3]. Parasite females were isolated with a paintbrush or forceps, washed with PBS pH 7.2 and processed for culture in the SGHP medium (Saline, Glucose, Human Plasma) described previously [4]. *Toxocara* eggs were harvested, concentrated by centrifugation, and incubated for one month until larvae developed, which were induced to hatch following the physiological method described elsewhere [16]. Larvae were purified with Lymphoprep and maintained in RPMI-1640 medium, to collect excretion-secretion antigens (L_2_TES) in a tube containing protease inhibitors cocktail (Sigma Aldrich, USA); subsequently they were concentrated by centrifugation in Amicon columns (10 KDa cutoff), quantified by the Bradford method, aliquoted and stored at −70°C until use [17].

### Monoclonal antibody (MoAb) production

Five female BALB/c mice were intraperitoneally inoculated with 500 live *Toxocara* larvae. Every two weeks a blood sample was collected from the tail vein; the sera were used to evaluate the immune response. Thirty days later, one mouse was euthanized, its spleen was isolated and the cells were fused with the mouse myeloma line X63Ag8.653 at a 5:1 ratio. Hybrid cells were selected following the standard method [18]. Chimeric cells secreting antibodies against *T. canis* larvae were selected. The cross-reactivity was tested using both excretion-secretion and somatic antigens of *T. canis* adult. Also *Toxocara cati, Ascaris suum, Trichinella spiralis*, *Ancylostoma caninum, Dipylidium caninum, Fasciola hepatica, Leishmania mexicana, Trypanosoma cruzi, Giardia intestinalis, Trichomonas vaginalis* and *Acanthamoeba spp.* antigens were tested. The controls were hyperimmune and preimmune sera from experimentally infected mouse. One MoAb (termed INP-1E4G4C2) was selected and cloned twice by limiting dilution [19]. The resultant antibody was purified using Montage Antibody Purification Kit with Prosep ®-G (Millipore, USA), typed [20] quantified and stored at −20°C until use.

### Polyclonal antibodies (PoAb) production

Two New Zealand rabbits were intraperitoneally injected with five thousand *T. canis* live larvae. To detect antibody increase, blood samples were obtained from the ear vein weekly. The rabbits were anesthetized and euthanized by bleeding when the titer was1:64,000. Serum was harvested and the IgG fraction was isolated with the Montage Antibody Purification Kit with Prosep ®-G (Millipore, USA), following the manufacturer’s instructions. The protein concentration was determined, and the antibodies were divided into aliquots and stored at −20°C until use.

### Determination of the optimal MoAb concentration for ELISA

**Figure 1:**
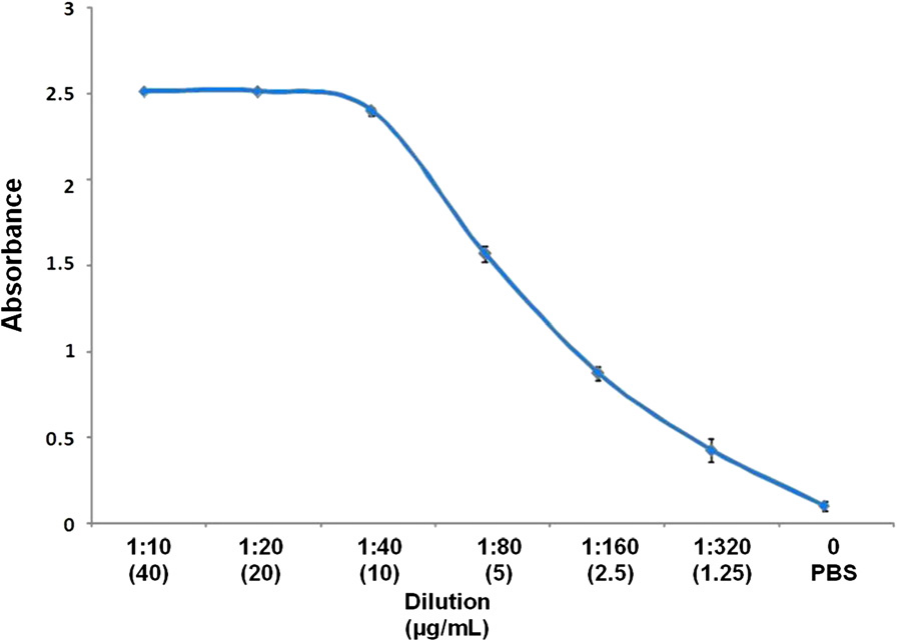
Determination of the optimal 1E4G4C2 MoAb concentration for the ELISA. 96-well polystyrene plate was coated with 0.0, 1.0, 2.5, 5.0, 10, 20 and 40 μg/mL of MoAb; non-specific binding sites were blocked with 1% skimmed milk; anti-mouse IgG-HRP conjugate was added at a 1:8,000 dilution and the reaction was revealed with chromogen/substrate solution. The results are shown as the arithmetic media ± standard deviation from three triplicate assays.

ELISA plates Immulon 2HB (Dynatech, USA) was coated with 100 μL/well of 0.0 to 40 μg/mL MoAb in borate buffer and incubated overnight at 4°C. The plates were then washed three times and non-specific binding sites were blocked with 1% skimmed milk in PBS-Tween 20 (0.05%) for 30 min at 37°C and then rinsed with 0.9%NaCl −0.05%Tween 20. An anti-mouse IgG-HRP conjugate (Sigma, A4416) was added at a 1:8,000 dilution in PBS-Tween 20, incubated for 2 h at 37°C and washed three times with 0.9%NaCl −0.05%Tween 20. The reaction was revealed with chromogen/substrate solution (0.05 M citrate/citric acid, 0.04 mg O-phenylenediamine and 0.12% H_2_O_2_). The reaction was stopped with H_2_SO_4_ and the absorbance was measured at 490 nm on an automatic ELISA reader Teca (Sunrise, Switzerland). The MoAb optimal concentration for the ELISA was 10.0 μg/mL (Figure 1).

### Sandwich ELISA standardization for L_2_TES detection

The L_2_TES capture was performed as follows: polystyrene wells were coated overnight at 4°C with 0.1, 1.0, 5.0, 10.0 and 25 μg/mL of PoAb in borate buffer (100 mM boric acid, 0.025 M sodium tetraborate, 75 mM, sodium chloride) pH 8. Unspecific sites were blocked for one hour at room temperature with 1% skimmed milk in PBS-Tween 20. L_2_TES were added at 0.0, and tenfold increment from 0.0001, until 10 μg/mL and incubated at 37°C for 2 h. Afterwards 10 μg/mL of the MoAb were added and incubated at room temperature. The anti-mouse IgG-HRP (1:8,000) was aggregated and incubated as described before. In every step, the plate was washed three times with NaCl -Tween 20.

### Detection of L_2_TES in human sera

We tested the method using 29 positive and 58 negative serum samples previously tested with a commercial kit (Sciemedx, USA) which detects antibodies against *T. canis* larvae antigens. A fraction of each sample was treated with EDTA to dissociate immune complexes; briefly, samples were diluted 1:1 with 1.0 M EDTA, pH 7.5, boiled for 10 minutes and centrifuged at 12,000 x g/5 minutes, and the supernatant was used in the test [21]. Another serum fraction was used diluted with PBS instead of EDTA. ELISA was performed with the PoAb at 5 μg/mL, 100 μL of sera with or without treatment with EDTA, the MoAb at 10 μg/mL and anti-mouse IgG-HRP diluted 1:8,000.

In all cases, the cut-off value was obtained adding three times the standard deviation to the mean absorbance value of the negative samples. The experiments were repeated three times.

### Western blot

Electrophoresis was performed in a Mini-Protean II (BioRad, USA) on 4-20% polyacrylamide slab gradient gels (BioRad,USA), with Kaleidoscope prestained standards (BioRad,USA) and 100 μg L_2_TES/well; electrophoresis was at 150 V for 2 h. Proteins were transferred to a PVDF membrane Immobilon (Millipore, USA) at 60 V for 1.5 h in Mini Trans-Blot (BioRad, USA). Nonspecific sites were blocked overnight with 5% skimmed milk in PBS-Tween 20 at 4°C. The blot was then incubated 2 h with INP-1E4G4C2 MoAb at room temperature and washed with PBS-Tween 20 (0.05%). The anti-mouse IgG-HRP conjugate, diluted 1:1,000 was then added and incubated for 2 h at room temperature. After three washes with PBS-Tween 20 (0.05%) and two with PBS, the substrate/chromogen solution (30 mg 3,3`-Diaminobenzidine, and 6 μL 30% H_2_O_2_ in 60 mL PBS) was added and the reaction was stopped with distilled water.

## Results

**Figure 2:**
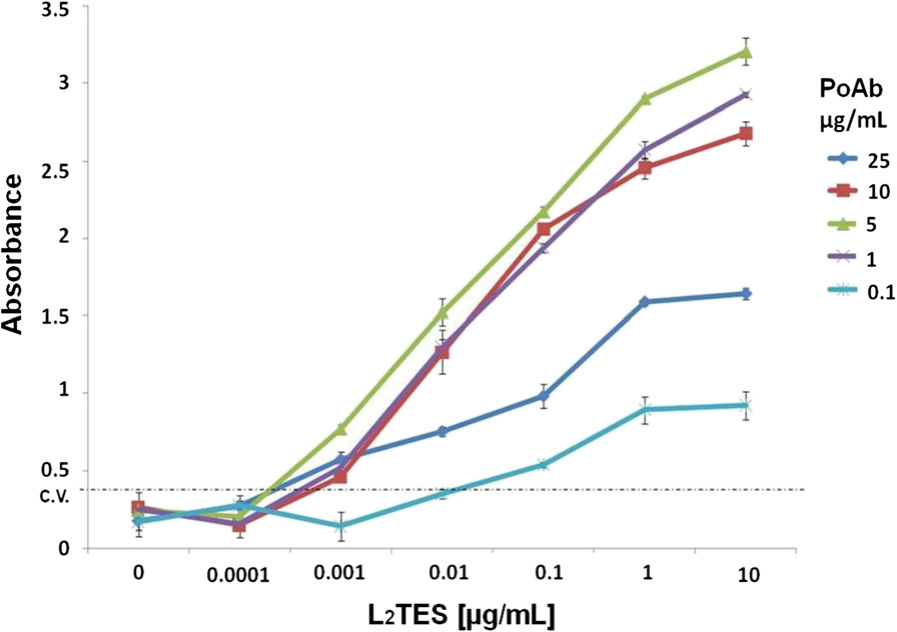
Determination PoAb optimal concentration to L_2_TES capture. 96-well polystyrene plate was coated with several concentrations of PoAb, non-specific binding sites were blocked with 1% skimmed milk; added different concentrations; 10 μg/mL of INP-1E4G4C2 MoAb, anti-mouse IgG-HRP conjugate at a 1:8,000 dilution and the reaction was revealed with chromogen/substrate solution. The reaction was stopped with H_2_SO_4_ and the absorbance was measured at 490 nm. The results are shown as the arithmetic media ± standard deviation from three triplicate assays.

The INP-1E4G4C2 is an IgG1 isotype MoAb that did not show cross-reactivity with other parasite antigens tested, including somatic antigens from *T. canis* adults. Only L_2_TES and larval somatic antigen gave high absorbance values; in contrast, the hyperimmune mouse serum recognized several parasites (Table 1). MoAb optimal concentration was 10 μg/mL because the absorbance inflection point was at 5.0 μg/mL (Figure 1). Sandwich ELISA standardization was with 5.0 μg/mL and 10 μg/mL of the polyclonal and monoclonal antibodies, and was detected since 440 pg/mL of L_2_TES (Figure 2).

**Table 1:** **Detection of cross-reactions of INP-1E4G4C2 MoAb against antigens from several parasites**

	**Absorbance at 490 nm**		
**Antigens**	**INP-1E4G4C2 MoAb**	**Hyperimmune serum**	**Preimmune serum**
*Toxocara canis* L_2_TES	2.5	2.5	0.116
*Toxocara canis* larvae somatic antigen	2.5	2.5	0.137
*Toxocara canis* ATES	0.27	2.5	0.146
*Toxocara canis* adult somatic antigen	0.287	1.842	0.204
*Toxocara cati*	0.201	1.089	0.085
*Ascaris suum*	0.285	0.825	0.099
*Ancylostoma caninum*	0.226	1.132	0.12
*Trichinella spiralis*	0.26	0.898	0.042
*Fasciola hepatica*	0.139	0.388	0.141
*Dipilidium caninum*	0.147	0.269	0.116
*Giardia intestinalis*	0.138	0.301	0.191
*Trypanosoma cruzi*	0.131	0.292	0.107
*Leishmania* spp	0.089	0.218	0.12
*Trichomonas vaginalis*	0.091	0.163	0.129
*Acantamoeba* spp	0.135	0.296	0.149

Cut-off: 0.35; L_2_TES: Larvae 2 *Toxocara* excretion-secretion antigens. ATES: Secretion excretion antigens from *Toxocara canis* adult.

**Figure 3:**
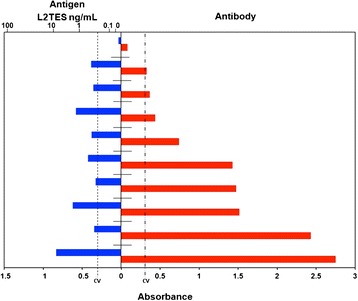
Detection of L_2_TES in human sera. 96-well polystyrene plate was coated with PoAb at 5 μg/mL, sera previously treatment with EDTA 100 μL, the MoAb at 10 μg/mL and anti-mouse IgG-HRP diluted 1:8,000.The reaction was revealed with chromogen/substrate solution. The reaction was stopped with H_2_SO_4_ and the absorbance was measured at 490 nm. The results are shown as the arithmetic media ± standard deviation from three triplicate assays.

With this technique we found from 470 pg/mL to 10 ng/mL of antigen in 9 out of 29 (sensitivity = 31%) positive sera previously diagnosed with a commercial kit that detects antibodies. In none of the 58 negative samples the antigen was detected (specificity = 100%) (Figure 3). We were able to detect antigens only in samples treated with EDTA.

**Figure 4:**
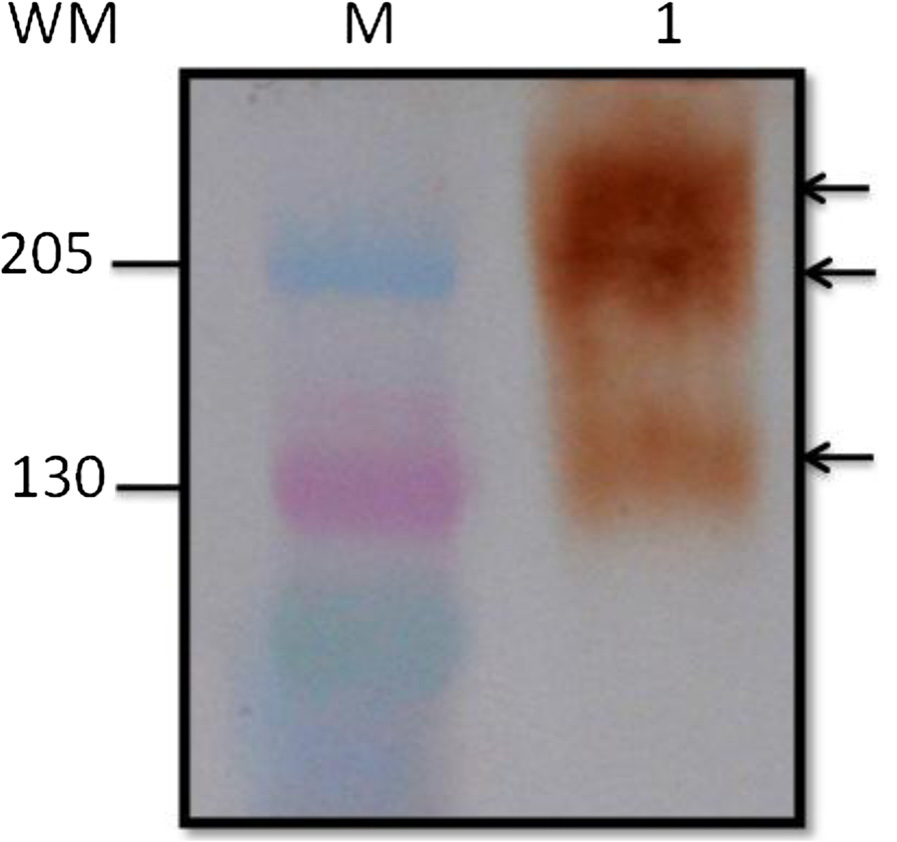
Western blot of L_2_TES with INP-1E4G4C2 MoAb Electrophoresis was performed on 4–20% polyacrylamide slab gradient gels former and separated at 150 V for 2 h. Proteins were transferred to a PVDF membrane immobilon. Nonspecific sites were blocked overnight with 5% skimmed milk in PBS-tween 20 at 4°C, incubated 2 h with INP-1E4G4C2 MoAb at room temperature and washed three times. Added and incubated for 2 h at room temperature with anti-mouse IgG-HRP conjugate (diluted 1:1,000) and washed three times; the substrate/chromogen solution was added and the reaction was stopped with distilled water. (M) kaleidoscope prestained standards. (1) 100 μg L_2_TES/well.

The western blot revealed that INP-1E4G4C2 MoAb recognized three bands of 130, 205 and >205 KDa, respectively (Figure 4).

## Discussion

*Toxocara larva migrans* diagnosis is not easy, because the methods are based on the detection of antibodies against the parasite*,* which do not determine the infection status and may give rise to false positive results, due to cross-reactions with other parasites, especially nematodes. With the intention to develop a technique for L_2_*T.canis* circulating antigens detection, we obtained one monoclonal antibody against L_2_*T. canis* antigens; which neither identified *T. canis* adult excretion-secretion and somatic antigens, nor presented cross-reactivity with other nematodes such as: *T. cati*, *A. suum*, *A. caninum* or *T. spiralis* (Table 1).

We used an approach of antigen capture by polyclonal antibodies instead of monoclonal, which gave higher analytical sensitivity (440 pg/mL of L_2_TES) than those described by Yokoi and Ishiyama, who used MoAbs to coat the plate and were able to capture antigen from 4.0 ng/mL and 5.0 ng/mL, respectively [14,15].

It has been suggested that several monoclonal antibodies can detect more than one band and that recognize more than one epitope [15]. The INP-1E4G4C2 MoAb detected three bands of L_2_TES, perhaps the capture of pg/mL quantities of L_2_TES was possible because the MoAb recognized several proteins which share epitopes. Yokoi [14] obtained a monoclonal antibody (IgG1) that did not cross-react with three parasites analyzed (*A. suum, D. immitis* and *T. canis* adult). Another study reported two monoclonal antibodies: one (IgM) that only identified *T. canis* excretion-secretion antigens, and other (IgG1) that distinguish *T. canis* and *T. cati* excretion-secretion antigens [13].

When we tested the method using antibody-positive samples, as defined by a commercial kit, we found circulating antigens in 31.0% of serum samples and none among 58 negative samples. These data suggest that 9 children had circulating antigens and perhaps larvae migration. It is likely that another 20 cases had antibodies against *Toxocara* from past infections, although it cannot be ruled out that they were individuals harboring other parasites, because the ELISA kit is unable to distinguish past infections from active infection and gives cross-reactions. Based on these data, we believe that the INP-1E4G4C2 MoAb could serve as a useful tool to demonstrate the presence of L_2_*T.canis* circulating antigens, and therefore, to establish the infection status of the host. Moreover, we considered it is necessary to test the sandwich ELISA in more samples from patients.

## Conclusions

With these tools we established a detection threshold as low as 440 pg/mL antigen. Monoclonal antibody is specific, and did not cross-react with antigens from other parasites. Detection of circulating antigens helps provide appropriate and timely treatment and prevents irreversible damage.

## Abbreviations

ATES: Secretion excretion antigens from *Toxocara canis* adult
CI: Confidence interval
CT: Covert toxocariasis
EDTA: Ethylenediaminetetraacetic acid
ELISA: Enzyme-Linked ImmunoSorbent Assay
EME: Eosinophilic meningo-encephalitis
H_2_O_2_: Hydrogen peroxide
H_2_SO_4_: Sulfuric acid
IgG-HRP: Horseradish peroxidase conjugated IgG
INP-1E4G4C2 MoAc: Monoclonal antibody produced in Instituto Nacional de Pediatría clone 1E4G4C2
L_2_*T.canis*: larvae 2 of *Toxocara canis*
L_2_TES: Larvae 2 *Toxocara* excretion-secretion antigens
MoAb: Monoclonal antibody
NaCl: Sodium chloride
NaCl Tween 20: Solution of sodium chloride with tween 20
OLM: Ocular *larva migrans*
PBS: Phosphate buffer solution
PBS-Tween 20: Phosphate buffer solution with tween 20
PoAb: Polyclonal antibodies
PVDF: Polyvinylidene difluoride
RPMI-1640: Roswell Park Memorial Institute medium 1640
SGHP: Medium of Saline, Glucose, Human Plasma
Tween 20: Polyoxyethylene Sorbitan Monolaurate
VLM: Visceral *larva migrans*
